# Self-reported physical and mental health status and quality of life in adolescents: a latent variable mediation model

**DOI:** 10.1186/1477-7525-8-17

**Published:** 2010-02-03

**Authors:** Richard Sawatzky, Pamela A Ratner, Joy L Johnson, Jacek A Kopec, Bruno D Zumbo

**Affiliations:** 1 School of Nursing, Trinity Western University, 7600 Glover Road, Langley, British Columbia (BC) V2Y 1Y1, Canada; 2 School of Nursing, University of British Columbia, 302-6190 Agronomy Road, Vancouver, BC V6T 1Z3, Canada; 3 School of Population and Public Health, University of British Columbia, 5804 Fairview Avenue, Vancouver, BC V6T 1Z3, Canada; 4 Department of ECPS, Measurement, Evaluation & Research Methodology, Scarfe Building, 2125 Main Mall, Vancouver, BC V6T 1Z4, Canada

## Abstract

**Background:**

We examined adolescents' differentiation of their self-reported physical and mental health status, the relative importance of these variables and five important life domains (satisfaction with family, friends, living environment, school and self) with respect to adolescents' global quality of life (QOL), and the extent to which the five life domains mediate the relationships between self-reported physical and mental health status and global QOL.

**Methods:**

The data were obtained via a cross-sectional health survey of 8,225 adolescents in 49 schools in British Columbia, Canada. Structural equation modeling was applied to test the implied latent variable mediation model. The Pratt index (*d*) was used to evaluate variable importance.

**Results:**

Relative to one another, self-reported mental health status was found to be more strongly associated with depressive symptoms, and self-reported physical health status more strongly associated with physical activity. Self-reported physical and mental health status and the five life domains explained 76% of the variance in global QOL. Relatively poorer mental health and physical health were significantly associated with lower satisfaction in each of the life domains. Global QOL was predominantly explained by three of the variables: mental health status (*d *= 30%), satisfaction with self (*d *= 42%), and satisfaction with family (*d *= 20%). Satisfaction with self and family were the predominant mediators of mental health and global QOL (45% total mediation), and of physical health and global QOL (68% total mediation).

**Conclusions:**

This study provides support for the validity and relevance of differentiating self-reported physical and mental health status in adolescent health surveys. Self-reported mental health status and, to a lesser extent, self-reported physical health status were associated with significant differences in the adolescents' satisfaction with their family, friends, living environment, school experiences, self, and their global QOL. Questions about adolescents' self-reported physical and mental health status and their experiences with these life domains require more research attention so as to target appropriate supportive services, particularly for adolescents with mental or physical health challenges.

## Background

Health researchers and providers increasingly recognize the importance of obtaining information about adolescents' perspectives of their quality of life (QOL) [1-10]. Several instruments have been developed for the measurement of adolescents' QOL to examine the impact of health care interventions, supportive services, and health promotion initiatives [e.g., [3,8,11,12]]. These instruments typically consist of subscales that represent experiences with various conditions in life (a.k.a. life domains) that are of general relevance to adolescents, including their perceived: (a) self (e.g., self-esteem), (b) relationships with friends and family, (c) experiences at school, and (d) living environment [13,14]. Often, the subscale scores are combined to obtain an overall, or global, QOL score. Other instruments include one or more general questions for the measurement of adolescents' global QOL in terms of their happiness or satisfaction with their lives. Despite the increasing availability of such instruments, the relationships among adolescents' self-reported health status (a.k.a. perceived or self-rated health status), their experiences with particular conditions in life, and their global QOL have not been examined extensively.

Several conceptual models have been developed to describe the relationships between health and QOL in *adult*s [15-22]. Most of these models emphasize assessing QOL from the perspective of the individual, and are based on the general proposition that alterations in health status affect other conditions in life (life domains), such as physical and psychological functioning, and social and environmental conditions, that are relevant to a person's QOL [e.g., [15,20-22]]. For example, Wilson and Cleary [15] introduced a very useful model of health and QOL wherein alterations in one's physiological condition (e.g., disease) result in physical and psychological changes that affect functional status, general health perceptions, and global or overall QOL. Concepts pertaining to characteristics of the individual (e.g., motivation and values) and characteristics of the environment (e.g., social support) are also taken into account. However, the relationship between *self-reported *health status and QOL is not expounded in the model; in particular, it is not clear how self-reported health status relates to other life domains relevant to QOL.

There is compelling empirical support for the associations between self-reported health status and QOL in general *adult *populations. A meta-analysis by Smith, Avis, and Assmann [23] showed that variation in QOL is explained by several life domains that are affected by differences in physiological health status (e.g., the presence of disease) and symptom severity. Their "model of the determinants of quality of life" (p. 448) is based on the proposition that the life domains mediate the associations between symptom severity and physiological health status, and QOL. Their regression analyses revealed that, relative to physical and social function, mental health status was by far the most important variable explaining QOL. Beckie and Hayduk [24], using structural equation modeling, similarly demonstrated that indicators of health status could be viewed as explanatory variables of QOL. Based on a study of adults who underwent coronary artery bypass graft surgery, they found that the eight health indicators measured by the Short-Form 36-item instrument (SF-36) [25] explained 67% of the variance in QOL, and that the effects of general health perceptions and mental health status were the most substantial. They concluded that "quality of life can be considered as a global personal assessment of a single dimension, which may be causally responsive to a variety of other distinct dimensions including dimensions such as health" (p. 281).

Several other researchers have examined the associations among self-reported health status, various life domains, and global QOL in adult populations [e.g., [26-28]]. However, information about these associations in *adolescent *populations is relatively sparse. The potential relevance of self-reported health status with respect to adolescents' QOL was shown in a study by Zullig et al. [29] who found that, in a sample of high-school students in South Carolina (U.S.A.), adolescents' self-reported health status was modestly correlated (*r *ranging from .09 to .22) with five life domains (satisfaction with family, friends, school, living environment, and self) and overall life satisfaction (*r *= .21). Other research has shown that adolescents' self-reported health status is associated with various health indicators, including physical activity, nutritional status, health-risk behavior, and physical disability [29-32]. Although these studies provide support for the measurement of adolescents' self-reported *general *health status, the differentiation of adolescents' self-reported *physical *and *mental *health status has not been extensively examined. Consequently, it is not known to what extent adolescents differentiate their physical and mental health status and whether this differentiation is relevant with respect to their global QOL and particular life domains.

### Study objectives

**Figure 1:**
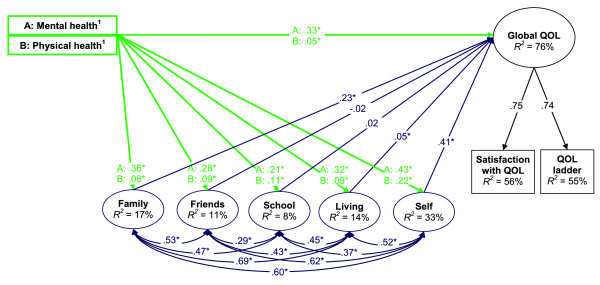
**Structural model of the relationships between self-reported physical and mental health status, domains of life satisfaction, and global QOL**. Notes: N = 6,932, WLSMV χ^2 ^(178) = 2,083.22 - 2,010.02, RMSEA = .049, CFI = .951. The variances of all latent factors were fixed at 1.0 for model identification. The measurement structures of the latent factors for each of the life domains are identical to those reported by Sawatzky et al. [37] (these are not shown here because of space limitations). All parameter values are standardized. The corresponding unstandardized parameters are provided in Table 4. ^1^Self-reported physical and mental health status were modeled as two ordinal variables with a latent factor that accounts for their correlation (not shown here). *p < .05.

We designed a study to: (a) validate adolescents' differentiation of their self-reported physical and mental health status and (b) examine the associations of these variables with global QOL and several relevant life domains, including adolescents' satisfaction with their family, friends, living environment, school, and self. With respect to the first objective, we hypothesized that, relative to one another, self-reported physical health status would be more strongly associated with physical activity, and self-reported mental health status with depressive symptoms. Drawing on the previously mentioned conceptual models and empirical research on health and QOL, we further sought to obtain information about (a) the relative importance of self-reported physical and mental health status with respect to adolescents' global QOL and several life domains and (b) the extent to which the relationships among self-reported physical and mental health statusand global QOL are mediated by the life domains (see Figure 1). Global QOL is viewed here as a unidimensional construct that pertains to individuals' satisfaction with, or appreciation of, their lives overall[18,30-32]. The life domains represent adolescents' satisfaction with various conditions in life that have the potential to contribute to their global QOL [33].

## Methods

### Sampling

The data were obtained via the British Columbia Youth Survey on Smoking and Health 2 (BCYSOSHII), a cross-sectional health survey that was conducted in 2004 to obtain information about tobacco dependence, drug and health-related behavior, and quality of life in adolescents in grades 7 to 12 in schools in the province of British Columbia (BC), Canada. The methods of this survey have been described in detail in several published studies [e.g., [34-39]]. The survey avoided two regional districts within the province that were known to have very low smoking prevalence rates so as to be cost-efficient in assembling a sample of adolescents that used tobacco (the primary purpose of the principal study). Nineteen of the 60 school districts in BC were sampled to achieve maximal geographic coverage of regional districts (remote and sparsely populated areas were not surveyed). Fourteen of the school district administrators provided permission for their schools to participate. This resulted in a sample of 89 eligible schools, ofwhich 49 (42 secondary schools, 5 alternative schools, and 2 middle schools) agreed to participate. Passive parental consent was obtained by providing parents with letters that informed them of the survey. Ethical approval was granted by the Behavioural Research Ethics Board of the University of British Columbia.

The survey questionnaire was administered by research assistants during class-time hours in pen and paper format (79.6%) or through a computer-based format (20.4%). The format was primarily determined by the availability of computers in the various schools. Less than 1% of the students refused to participate and the response rate within schools was 84%, on average (non-response was mostly due to student absenteeism) [34,36]. The resulting sample consisted of 8,225 adolescents (smokers and non-smokers).

### Measurement

Self-reported physical and mental health status were measured using two questions, "How would you rate your physical health?" and "How would you rate your emotional or mental health?" with the following response options, which were taken from the general health status question of the SF-36 [25] and which are widely used in the national population health surveys of many countries: "excellent" (coded as "5"), "very good," "good," "fair," or "poor" (coded as "1"). The validity of measuring adolescents' self-reported *general *health status in this manner is supported by observed associations with various other health status indicators, including physical activity, nutrition, health-risk behavior, and physical disability [40-43]. Study findings have consistently revealed that a relative increase in adolescents' self-reported general health status is associated with less health-risk behavior and fewer days of limited activity [41,43].

To validate adolescents' differentiation of their *physical *and *mental *health status, we examined the relative importance of these variables with respect to depressive symptoms and the frequency of physical activities. *Depressive symptoms *were measured using 12 items from the Center of Epidemiologic Studies Depression Scale (CES-D) [44]. The adolescents were asked: "How often have you felt or behaved in the following manner in the past week (7 days)?" (e.g., "hopeful about the future," "happy," "lonely," "sad"). The CES-D provides four response options ranging from "rarely or none of the time (less than one day)" (coded as "0") to "most or all of the time (5-7 days)" (coded as "3"). The total score, with a possible range of 0 (no depressive symptoms) to 36, was used in the analysis. The estimated reliability of the 12 items is .87 in this sample (based on the ordinal Cronbach alpha reliability estimate [45]). *Physical activity *was measured using the following question adapted from several large surveys (e.g., The USA Youth Risk Behavior Survey [46] and The Ontario Drug Use Survey [47]): "On how many of the last 7 days did you exercise or participate in sports activities for at least 20 minutes that made you sweat and breathe hard? If none, enter '0' days. Please include activities such as basketball, jogging, swimming, cross-country skiing, hockey, or dance, that you participated in either at school or outside of school."

An abridged version of Huebner's *Multidimensional Students' Life Satisfaction Scale *(MSLSS) [48] was used to measure adolescents' satisfaction with five life domains, including their family (4 items), school (4 items), living environment (2 items), friends (4 items) and self (4 items) [37]. The original MSLSS consists of 40 items, of which 10 are negatively worded. The psychometric analyses reported by Sawatzky et al. [37] revealed that the adolescents may not have interpreted and responded to all items in the same way. There were inconsistencies in the responses to the negatively worded items and several other items. An abridged 18-item version was developed by identifying those items that were found to be most invariant (all positively worded). Confirmatory factor analyses (CFA) provided support for its construct validity when allowing for a few theoretically defensible modifications [37]. The same measurement structure was used to represent the five life domains as latent factors in the study reported herein. The ordinal Cronbach alpha reliability estimates [45] of the abridged subscales with four items were ≥ .80 in this sample. A six-point ordinal response format (with response options ranging from "strongly disagree" (coded as "1") to "strongly agree" (coded as "6")) was used [49].

**Figure 2:**
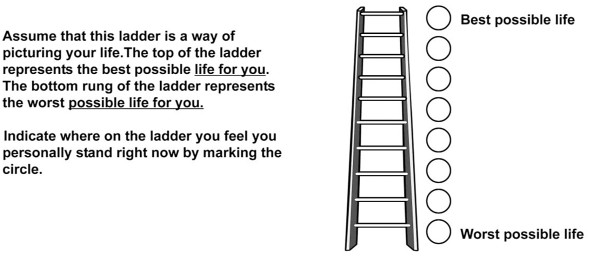
**Quality of life ladder**. Notes: Derived from Cantril's self-anchoring ladder [50]. An error resulted in 8 rungs being presented in the paper-based version whereas 10 rungs were presented in the computer version. To remedy this, we rescaled the QOL-ladder for the computer- and paper-based versions to their common denominator by multiplying the computer-based version of the QOL-ladder by 0.8 and rounding the resulting scores to zero decimals.

Global QOL was measured with two items. The adolescents were asked to appraise their QOL using a picture of an eight-rung ladder (Cantril's self-anchoring ladder [50], referred to here as the QOL-ladder) (see Figure 2). The bottom run was coded as "1" and the top as "8". The adolescents also were asked to rate their agreement with the statement, "I am satisfied with my quality of life" with four response options ranging from "strongly disagree" (coded as "1") to "strongly agree" (coded as "4"). General questions of this nature, including Cantril's self-anchoring ladder, have been widely used in surveys for the measurement of various concepts such as global QOL [51-53]. A latent factor explaining the variance in both of these variables was used to represent global QOL.

The adolescents were asked to indicate their age and sex, and to answer several questions about their ethnic identity and living arrangements. Ethnic identity was determined by asking, "How would you describe yourself?" The 12 response options were adapted from Statistics Canada's [54] classification of "visible minorities" (e.g., "white/Caucasian," Aboriginal/First Nation, Chinese, South East Asian). The adolescents selected one or more responses, which were subsequently grouped as: "white/Caucasian," Asian (including Chinese, Japanese, Korean, South East Asian, and Filipino), Aboriginal/First Nation, and "other." With respect to their living arrangements, the adolescents were asked, "Which parent or parents do you currently live with most of the time?" with eight response options (i.e., mother, father, step-mother, step-father, guardian(s), foster parent(s), grandparent(s), and other please specify).

### Statistical methods

Structural equation modeling was used to examine the hypothesized relationships by fitting a latent variable mediation model to the sample data (see Figure 1). The variances of the latent factors were specified to equal one to avoid indeterminancy and to set the metric of the latent factors [55]. Polychoric correlations were used to avoid obtaining biased parameter estimates due to the ordinal distributions of the observed variables [56-58]. The MPlus 5.2 software [59] was used to estimate the model parameters by specifying a probit link function and using a mean and variance adjusted weighted-least squares estimation method (WLSMV) suitable for ordinal data [60]. Model fit was evaluated with several global fit indices, and by comparing the differences between the implied and the observed polychoric correlation matrices. Adequate model fit was defined by a root mean square error of approximation (RMSEA) of < .06 [61] and a comparative fit index (CFI) of ≥ .95 [62]. In addition, the pattern and magnitudes of the residual correlations were examined to locate any specific areas of misfit [63,64]. The percentage of residual correlations with absolute values greater than .10 is provided as a summary of this direct comparison.

The relative importance of the explanatory variables was determined by the Pratt index (*d*) [65], which quantifies each variable's contribution to the explained variance (irrespective of the magnitude of the *R*-squared), measured as a percentage. The extent to which the two relationships between global QOL and physical and mental health status were mediated by the life domains was evaluated as the division of the indirect-effects (mediated by the life domains) and the total effect (the sum of the direct- and indirect-effects for the associations between global QOL and physical and mental health status), expressed as a percentage [66]. The standard error of the indirect effects was calculated using the Delta method, which is similar to the approach used in the Sobel test [67].

Of the 8,225 adolescents, 920 did not provide responses to any of the MSLSS questions. The analysis was limited to those who responded to the global QOL items, the items measuring mental or physical health status, and at least one of the MSLSS items (*N *= 6,932). Multiple imputation (MI) [68] was used to impute any remaining missing responses (2.5% imputed data). The results were compared with those obtained using MI for the subsample of adolescents who provided a value for at least one of the analysis variables (*N *= 8,174; 13.9% imputed data). The SAS 9.2 software package [69] was used to create 10 imputed datasets for the MI analyses, following the guidelines offered by Allison [70] and Enders [71], to assess convergence and to incorporate auxiliary variables (i.e., demographic variables (sex, ethnicity, school grade), symptoms of depression, and two variables pertaining to the adolescents' experiences at school).

## Results

### Sample description

The sample consisted of an approximately equal proportion of boys and girls in grades 7 through 12 (see Table 1). The average age was 15.2 years (SD = 1.5, *n *= 8,054) with 7,964 adolescents being between 12 and 18 years. Although most of the adolescents who identified their ethnicity (*n *= 7,882) self-identified as "white/Caucasian" (72.6%), the sample also included Aboriginal adolescents (16.5%), Asian adolescents (Chinese, Japanese, Korean, Filipino, or South-East Asian) (5.8%), and adolescents belonging to one or more other groups (5.1%). A sizeable percentage (17.3% of 7,994 adolescents) indicated regularly speaking a language other than English, and 6.9% of 8,058 reported being born in a country other than Canada.

**Table 1:** Sample description

Variable	Percentage
Minority status (*N *= 7,882)	
No, "white"	72.6%
Yes, Asian	5.8%
Yes, Aboriginal	16.5%
Other or mixed	5.1%
Sex (*N *= 8,163)	
Male	49.8%
Female	50.2%
Grade (*N *= 8,074)	
Grades 7 or 8	23.2%
Grade 9	19.4%
Grade 10	23.7%
Grade 11	21.1%
Grade 12 or "other"	12.6%
Living arrangements (*N *= 7,582)	
Lives with mother *and *father	59.9%
Lives with mother *and not *father	25.7%
Lives with father *and not *mother	7.8%
Does *not *live with mother or father	6.7%
Satisfied with quality of life (*N = *7,606)	
Strongly disagree	4.6%
Disagree	13.0%
Agree	52.7%
Strongly agree	29.6%

Percentages may not sum to exactly 100% due to rounding.

Most of the adolescents agreed or strongly agreed to being satisfied with their QOL (82.3% of 7,606 adolescents) (see Table 1). The mode of the QOL-ladder responses was at level 6 of 8 rungs (36.7%), with 11.9% of the adolescents reporting the best possible life, and 14.0% providing a rating at or below the middle of the scale (≤ 4) (*n *= 7,675).

### The measurement of self-reported physical and mental health status

The joint- and marginal-distributions of self-reported physical and mental health status are provided in Table 2. The corresponding conditional distributions provide support for adolescents' ability to differentiate these variables. For example, 9.5% of the adolescents who rated their physical health as good or better rated their mental health as fair or poor, and 5.3% of the adolescents who rated their mental health as good or better rated their physical health as fair or poor. The polychoric correlation was .55, indicating a shared variance of only 30% among these two (underlying) variables. With respect to the differentiation of mental and physical health status (see Table 3), we found that 94% (*d*, Pratt Index) of the explained variance in depressive symptoms (*R*^2 ^= 35.5%) could be attributed to mental health status (the remaining 6% was attributed to physical health status). Conversely, relative to self-reported physical health status, self-reported mental health status accounted for only 18% of the explained variance in physical activity (*R*^2 ^= 7.7%) (see Table 3).

**Table 2:** Joint and marginal distributions of self-reported physical and mental health status

	Mental health					
		
Physical health	Excellent	Very good	Good	Fair	Poor	Total
excellent	1,367 (17.6%)	502 (6.4%)	156 (2.0%)	49 (0.6%)	30 (0.4%)	2,104 (27.0%)
very good	876 (11.3%)	1,333 (17.1%)	563 (7.2%)	160 (2.1%)	39 (0.5%)	2,971 (38.2%)
good	316 (4.1%)	615 (7.9%)	739 (9.5%)	315 (4.0%)	82 (1.1%)	2,067 (26.6%)
fair	53 (0.7%)	89 (1.1%)	181 (2.3%)	167 (2.1%)	52 (0.7%)	542 (7.0%)
poor	15 (0.2%)	7 (0.1%)	18 (0.2%)	25 (0.3%)	35 (0.5%)	100 (1.3%)

total	2,627 (33.7%)	2,546 (32.7%)	1,657 (21.3%)	716 (9.2%)	238 (3.1%)	7,784

All percentages are of the total sample.

**Table 3:** Relationships between self-reported physical and mental health status and depressive symptoms and frequency of physical activity

Variable	*b*	*SE b*	β	*r*	*d*
Depressive symptoms (*N *= 7,985; *R*^2 ^= 35.5%)					

Physical health	-0.46	0.09	-.06	-.33	6%
Mental health	-3.73	0.08	-.56	-.59	94%

Physical activity (*N *= 7,033; *R*^2 ^= 7.7%)					

Physical health	0.68	0.04	.24	.27	82%
Mental health	0.18	0.03	.07	.19	18%

Notes: *r *= bivariate polyserial correlations, *d *= Pratt Index. All parameter estimates are statistically significant (*p *< .05).

### The associations between health status and quality of life

The hypothesized model with the life domains operating as mediators of the relationships between self-reported physical and mental health status and global QOL resulted in acceptable overall fit (WLSMV χ^2 ^ranging from 2,083.22 to 2,010.02 for the 10 MI datasets (*N *= 6,932), RMSEA = .049, CFI = .951, residual correlations ranging from -.07 to .07) (see Figure 1). Satisfaction with family, friends, school, living-environment, and self, and self-reported physical and mental health status explained 76.1% of the variance in global QOL.  Although self-reported physical and mental health status were bivariately significantly correlated with global QOL (*r *= .49 and .70, respectively), their associations were substantially smaller, albeit statistically significant, in the multivariate model (see Table 4). The life domains also were bivariately significantly correlated with global QOL. However, relatively small and statistically non-significant regression coefficients were obtained for satisfaction with friends and satisfaction with school in the multivariate model (see Table 4). These variables accounted for less than 2% (*d*, Pratt Index) of the explained variance relative to the other variables in the model (see Table 4). Global QOL was mostly explained by satisfaction with self (*d *= 42%), self-reported mental health status (*d *= 30%), and satisfaction with family (*d *= 20%). Self-reported physical health status accounted for only 3% of the explained variance.

**Table 4:** Relative importance of variables explaining global QOL

Variable	*b*	*SE B*	β	*r *	*d*
Family	0.29*	0.03	.23*	.66*	20%
Friends	-0.02*	0.02	-.02*	.51*	0%
School	0.02*	0.01	.02*	.40*	1%
Living environment	0.05*	0.02	.05*	.56*	4%
Self	0.62*	0.03	.41*	.78*	42%
Mental health	0.26*	0.01	.33*	.70*	30%
Physical health	0.04*	0.01	.05*	.49*	3%

Notes: *r *= bivariate correlation with the latent global QOL variable, *d *= Pratt index. *N *= 6,932. *R*^2 ^= 76%. * *p < .05*.

Self-reported physical and mental health status were significantly correlated with each of the life domains (*r*_*physical health *_ranging from .22 to .45; *r*_*mental health *_ranging from .27 to .54), and they predominantly explained satisfaction with self (*R*^2 ^= 33.0%), and, to a lesser extent, satisfaction with family (*R*^2 ^= 16.9%), friends (*R*^2 ^= 11.3%), and living environment (*R*^2 ^= 14.2%) (see Table 5). Only 7.9% of the variance in satisfaction with school was explained by self-reported physical and mental health status. Relative to self-reported physical health status, most of the variance in each of the life satisfaction dimensions could be attributed to the adolescents' self-reported mental health status (*d *ranging from 68% to 87% for each of the life domains) (see Table 5).

**Table 5:** Relative importance of variables explaining the dimensions of life satisfaction

Variable	*b*	*SE B*	β	*r*	*d*
Explaining satisfaction with family (*R*^2 ^= 16.9%)					

Physical health	0.05	0.01	.08	.27	13%
Mental health	0.23	0.01	.36	.41	87%

Explaining satisfaction with friends (*R*^2 ^= 11.3%)					

Physical health	0.07	0.02	.09	.24	19%
Mental health	0.24	0.01	.28	.33	81%

Explaining satisfaction with school (*R*^2 ^= 7.9%)					

Physical health	0.09	0.01	.11	.22	32%
Mental health	0.17	0.01	.21	.27	68%

Explaining satisfaction with living environment (*R*^2 ^= 14.2%)					

Physical health	0.07	0.01	.09	.26	16%
Mental health	0.28	0.02	.32	.37	84%

Explaining satisfaction with self (*R*^2 ^= 33.0%)					

Physical health	0.11	0.01	.22	.45	30%
Mental health	0.22	0.01	.43	.54	70%

Notes: *r *= bivariate correlation with the latent variable, *d *= Pratt index. *N *= 6,932. All parameter estimates are statistically significant (*p *< .05).

The parameters for the relationships between physical and mental health status, the life domains, and global QOL were used to determine the magnitude of the total and the indirect relationships between physical and mental health status and global QOL as mediated by each of the life domains (see Table 6). The standardized total effect on global QOL was larger for self-reported mental health status (β = .61), while adjusting for self-reported physical health status, than for self-reported physical health status (β = .17), while adjusting for self-reported mental health status. These relationships were partially mediated by the life domains (67.8% total mediation for physical health and 45.4% total mediation for mental health status). The relationships between the two health status variables and global QOL were primarily mediated by satisfaction with self (54.0% mediation for self-reported physical health and 29.1% mediation for self-reported mental health) and, to a lesser extent, by satisfaction with family (10.8% mediation for self-reported physical health and 13.7% mediation for self-reported mental health).

**Table 6:** Mediation effects for physical and mental health status and global QOL

	Effect of self-reported physical health status on global QOL			Effect of self-reported mental health status on global QOL		
	
Mediating variable	***B***_**indirect**_	*SE B*	% mediation	***B***_**indirect**_	*SE B*	% mediation
Family^1^	0.01	0.00	10.8%	0.07	0.01	13.7%
Friends^1^	-0.00	0.00	-1.0%	-0.00	0.00	-0.8%
Living^1^	0.00	0.00	2.8%	0.01	0.01	2.8%
School^1^	0.00	0.00	1.2%	0.00	0.00	0.6%
Self^1^	0.07	0.01	54.0%	0.14	0.01	29.1%

Total indirect effects^2^	0.08		67.8%	0.22		45.4%

Notes: Degree of mediation attributed to each satisfaction variable was calculated as the indirect effect for that variable divided by the total effect for physical or mental health status. *N *= 6,932.

^1 ^Indirect effect of physical or mental health status on global quality of life as mediated by one of the life domains.

^2 ^Sum of all indirect effects for physical and mental health status explaining global quality of life.

## Discussion

This study provides support for (a) the notion that adolescents can differentiate between physical and mental health when they provide reports of their health status and (b) the relevance of this differentiation with respect to five life domains and global QOL. The results revealed that relatively poorer self-reported physical and mental health status were significantly associated with lower global QOL and lower satisfaction with each of the life domains. The adolescents' global QOL was predominantly explained by mental health status and by their satisfaction with self and family. Satisfaction with self and family were the main mediating variables for the relationships between mental health status (45.4% total mediation) and physical health status (67.8% total mediation) and global QOL.

Other studies have shown that self-reported *general *health status is significantly associated with health-promoting and health-risk behavior [40-43] and with various life domains and global QOL [29]. Our study contributes to this area of research by providing preliminary support for the validity and the relevance of distinguishing between adolescents' self-reports of their physical and mental health status. The findings suggest that, relative to one another, self-reported mental health status is more strongly associated with depressive symptoms and physical health status with physical activity. Although further research is needed to examine the validity and relevance of these variables with respect to other research objectives (e.g., their associations with particular health-risk behavior), the current findings suggest that the use of two self-report items for the measurement of adolescents' physical and mental health status could contribute valuable information in population-based adolescent health surveys.

There were substantial differences in the associations between self-reported physical and mental health status and adolescents' global QOL and the five life domains. The correlations with self-reported mental health status were greater than were those with physical health status. This finding is congruent with a study by Zullig et al. [29] who found that, relative to the self-reported number of days with poor physical health, the number of poor mental health days was more strongly correlated with adolescents' overall life satisfaction (*r *= -.27 versus -.15) and their satisfaction with their family (*r *= -.25 versus -.14), friends (*r *= -.10 versus -.07), living environment (*r *= -.15 versus -.10), school (*r *= -.15 versus -.12) and their self perception (*r *= -.29 versus -.21). However, in our study, the correlations with global QOL (*r*_*physical health *_= .49; *r*_*mental health *_= .70), and each of the life domains (*r*_*physical health *_ranging from .22 to .45; *r*_*mental health *_ranging from .27 to .54;) were relatively stronger. It is possible that the measurement of self-reported physical and mental health status (rather than the number of poor physical and mental health days), and the use of the abridged MSLSS for the five life domains (rather than the use of single items for each of the life domains), resulted in greater sensitivity to detect these associations.

In addition to these bivariate associations, our study provides information about the relative importance of self-reported physical and mental health status and the five life domains in explaining global QOL in adolescents. The results revealed that self-reported physical health status contributed minimally to global QOL when controlling for the other variables in the model; its association with global QOL was significantly confounded by self-reported mental health status and the five life domains. Self-reported mental health status was relatively more important with respect to each of the life domains, and it was the second most important explanatory variable for global QOL. These findings provide support for attending to the mental health needs of adolescents.

With respect to each of the life domains, we found that most of the variance in global QOL could be attributed to the adolescents' satisfaction with themselves and their families. The associations between satisfaction with friends and school and global QOL were not statistically significant in the multivariate model. These findings are congruent with a study by Gilman [72] who found that, in a sample of 321 high-school students in a Southeastern US state, the associations between satisfaction with friends and school and global QOL were relatively small when controlling for the other life domains. It is possible that adolescents' satisfaction with their friends and their school is associated with their satisfaction with their family, and that these associations are therefore confounded in the multivariate model. This is an important area for further study.

An important theoretical conclusion to be drawn from these findings is that self-reported physical and mental health status and the life domains can be viewed as conditions that contribute to global QOL in adolescents. These relationships are fundamentally different from those implied by the common practice of deriving global QOL scores from the combined scores of particular life domains. Many multidimensional instruments designed to measure QOL are based on the assumption that scores pertaining to various life domains can be combined so as to obtain an overall (general) QOL score. For instance, it has been argued that an overall QOL score could be obtained by averaging the scores of the five life domain subscales of the MSLSS [49,73,74]. The theoretical premise of this approach is that the experiences in the various life domains reflect, or *arise from*, a common source, labeled global QOL. This premise is not congruent with the previously noted conceptualization of life domains as conditions that *contribute to *QOL. Our analyses demonstrate a different approach that is congruent with the conceptualization of QOL as a global concept that is partially explained by various contributing conditions, such as health status and people's experiences with various other aspects of life (life domains) [23,24,26-28,32,33].

There are several limitations to this study that must be taken into account. First, the cross-sectional nature of this analysis does not warrant conclusive statements about the causal nature of the relationships. Claims pertaining to the direction and causal nature of these relationships require further investigation. Second, although care was taken to limit the bias that may have resulted from missing data, it is possible that there were systematic differences between the adolescents who did not respond to all the items in comparison with those who did. Third, it is possible that different magnitudes of the observed relationships would be obtained in different populations, or groups, of adolescents. For instance, the relative importance of the life domains may be different for boys and girls or for adolescents from different age-groups or cultural or socio-economic backgrounds. We therefore recommend further research to examine the differences in the magnitudes of the associations between health status, important life domains, and global QOL in different adolescent populations.

## Conclusions

This study provides support for a conceptual model of self-reported physical and mental health status and several life domains that contribute to adolescents' global QOL. Support is also provided for the use of distinct items to measure self-reported physical and mental health status in adolescent population health surveys. Mental health status and, to a lesser extent, physical health status were associated with significant differences in the adolescents' appraisals of their family, friends, living environment, school experiences, self, and their global QOL. Questions pertaining to these important life domains require more attention in health assessments and in population health research so as to target appropriate supportive services for adolescents with mental or physical health challenges.

## List of abbreviations

BCYSOSH II: British Columbia Youth Survey on Smoking and Health 2; MSLSS: Multidimensional Students' Life Satisfaction Scale; QOL: Quality of life; β: Standardized regression coefficient; b: Unstandardized regression coefficient; CFI: Comparative fit index; *d*: Pratt index; LR: Likelihood ratio; OR: Odds ratio; RMSEA: Root mean square error of approximation; *r*: Correlation; SE: Standard error; SD: Standard deviation; WLSMV: Weighted least squared, mean and variance adjusted.

## Competing interests

The authors declare that they have no competing interests.

## Authors' contributions

RS and PR designed and carried out the statistical analyses and drafted the manuscript. JJ was the principal investigator for the British Columbia Youth Survey on Smoking and Health 2. All authors contributed substantially to the design of the study, the interpretation of the results, and the editing of the manuscript. All authors read and approved the final manuscript.

## References

[B1] Kaplan RM Drotar D Implication of quality of life assessment in public policy for adolescent health Measuring Health-Related Quality of Life in Children and Adolescents 1998 Mahwah, NJ: Lawrence Erlbaum 63 84

[B2] Raphael D Determinants of health of North-American adolescents: evolving definitions, recent findings, and proposed research agenda J Adolesc Health 1996 19 6 16 10.1016/1054-139X(95)00233-I 8842855

[B3] Raphael D Brown I Rukholm E Hill-Bailey P Adolescent health: moving from prevention to promotion through a quality of life approach Can J Public Health 1996 87 81 83 8753631

[B4] Dannerbeck A Casas F Sadurni M Coenders G Quality-of-Life Research on Children and Adolescents 2004 Dordrecht, Netherlands: Kluwer

[B5] Topolski TD Patrick DL Edwards TC Huebner CE Connell FA Mount KK Quality of life and health-risk behaviors among adolescents J Adolesc Health 2001 29 426 435 10.1016/S1054-139X(01)00305-6 11728892

[B6] Wallander JL Schmitt M Koot HM Quality of life measurement in children and adolescents: issues, instruments, and applications J Clin Psychol 2001 57 571 585 10.1002/jclp.1029 11255207

[B7] Topolski TD Edwards TC Patrick DL Toward youth self-report of health and quality of life in population monitoring Ambul Pediatr 2004 4 387 394 10.1367/A03-131R.1 15264944

[B8] Huebner ES Valois RF Suldo SM Smith LC McKnight CG Seligson JL Zullig KJ Perceived quality of life: a neglected component of adolescent health assessment and intervention J Adolesc Health 2004 34 270 278 15040996 10.1016/j.jadohealth.2003.07.007

[B9] Koot HM Wallander JL Quality of Life in Child and Adolescent Illness: Concepts, Methods and Findings 2001 Hove, East Sussex: Brunner-Routledge

[B10] Huebner ES Nagle RJ Suldo S Quality of life assessment in child and adolescent health care: the Multidimensional Students' Life Satisfaction Scale (MSLSS) Social Indicators Research Series 2003 20 179 189

[B11] Patrick DL Edwards TC Topolski TD Adolescent quality of life, part II: initial validation of a new instrument J Adolesc 2002 25 287 300 10.1006/jado.2002.0471 12128039

[B12] Bradford R Rutherford DL John A Quality of life in young people: ratings and factor structure of the Quality of Life Profile-Adolescent Version J Adolesc 2002 25 261 274 10.1006/jado.2002.0469 12128037

[B13] Edwards TC Huebner CE Connell FA Patrick DL Adolescent quality of life, part I: conceptual and measurement model J Adolesc 2002 25 275 286 10.1006/jado.2002.0470 12128038

[B14] Huebner ES Preliminary development and validation of a multidimensional life satisfaction scale for children Psychol Assess 1994 6 149 158 10.1037/1040-3590.6.2.149

[B15] Wilson IB Cleary PD Linking clinical variables with health-related quality of life. A conceptual model of patient outcomes J Am Med Assoc 1995 273 59 65 10.1001/jama.273.1.59 7996652

[B16] Ferrans CE Lipscomb J, Gotay CC, Snyder C Definitions and conceptual models of quality of life Outcomes Assessment in Cancer: Measures, Methods, and Applications 2005 Cambridge, NY: Cambridge University Press 14 30

[B17] Vallerand AH Payne JK King CR, Hinds PS Theories and conceptual models to guide quality of life research Quality of Life From Nursing and Patient Perspectives: Theory, Research, Practice 2003 2 Sudbury, MA: Jones and Bartlett 45 64

[B18] Nordenfelt L Quality of Life, Health and Happiness 1993 Aldershot, England: Avebury

[B19] Michalos AC Multiple discrepancies theory (MDT) Soc Indic Res 1985 16 347 413 10.1007/BF00333288

[B20] Burckhardt CS The impact of arthritis on quality of life Nurs Res 1985 34 11 16 10.1097/00006199-198501000-00003 3844156

[B21] Padilla GV Grant MM Quality of life as a cancer nursing outcome variable ANS Adv Nurs Sci 1985 8 45 60 3933413 10.1097/00012272-198510000-00007

[B22] Patrick DL Chiang YP Measurement of health outcomes in treatment effectiveness evaluations: conceptual and methodological challenges Med Care. 2000 38 9 Suppl II14 25 10982087 10.1097/00005650-200009002-00005

[B23] Smith KW Avis NE Assmann SF Distinguishing between quality of life and health status in quality of life research: a meta-analysis Qual Life Res 1999 8 447 459 10.1023/A:1008928518577 10474286

[B24] Beckie TM Hayduk LA Using perceived health to test the construct-related validity of global quality of life Soc Indic Res 2004 65 279 298 10.1023/B:SOCI.0000003800.31366.73

[B25] Ware JE Snow KK Kosinski M Gandek B SF-36 Health Survey: Manual and Interpretation Guide 1993 Boston, MA: The Health Institute, New England Medical Center

[B26] Michalos AC Hubley AM Zumbo BD Hemingway D Health and other aspects of the quality of life of older people Soc Indic Res 2001 54 239 274 10.1023/A:1011045307643

[B27] Michalos AC Thommasen HV Read R Anderson N Zumbo BD Determinants of health and the quality of life in the Bella Coola Valley Soc Indic Res 2005 72 1 50 10.1007/s11205-004-4512-5

[B28] Michalos AC Zumbo BD Hubley A Health and the quality of life Soc Indic Res 2000 51 245 286 10.1023/A:1007010401301

[B29] Zullig KJ Valois RF Drane JW Adolescent distinctions between quality of life and self-rated health in quality of life research Health Qual Life Outcomes 2005 3 64 10.1186/1477-7525-3-64 16248897 PMC1280929

[B30] Veenhoven R The four qualities of life J Happiness Stud 2000 1 1 39 10.1023/A:1010072010360

[B31] Musschenga AW The relation between concepts of quality-of-life, health and happiness J Med Philos 1997 22 11 28 9095459 10.1093/jmp/22.1.11

[B32] Beckie TM Hayduk LA Measuring quality of life Soc Indic Res 1997 42 21 37 10.1023/A:1006881931793

[B33] Campbell A Converse P Rodgers WL The Quality of American Life 1976 New York: Sage

[B34] Tu AW Ratner PA Johnson JL Gender differences in the correlates of adolescents' cannabis use Subst Use Misuse 2008 43 1438 1463 10.1080/10826080802238140 18696378 PMC2562034

[B35] Richardson CG Johnson JL Ratner PA Zumbo BD The influence of web- versus paper-based formats on the assessment of tobacco dependence: evaluating the measurement invariance of the Dimensions of Tobacco Dependence Scale Subst Abuse 2009 3 1 14 10.4137/sart.s960 PMC3864853 24357926

[B36] Richardson CG Johnson JL Ratner PA Zumbo BD Bottorff JL Shoveller JA Prkachin KM Validation of the Dimensions of Tobacco Dependence Scale for adolescents Addict Behav 2007 32 1498 1504 10.1016/j.addbeh.2006.11.002 17175114

[B37] Sawatzky R Ratner PA Johnson JL Kopec J Zumbo BD Sample heterogeneity and the measurement structure of the Multidimensional Students' Life Satisfaction Scale Soc Indic Res 2009 94 273 296 10.1007/s11205-008-9423-4 PMC5112029 27867252

[B38] Okoli CT Richardson CG Ratner PA Johnson JL An examination of the smoking identities and taxonomies of smoking behaviour of youth Tob Control 2008 17 151 158 10.1136/tc.2007.021683 18270230

[B39] Sawatzky R Gadermann A Pesut B An investigation of the relationships between spirituality, health status and quality of life in adolescents Applied Research in Quality of Life 2009 4 5 22 10.1007/s11482-009-9065-y

[B40] Wade TJ Vingilis E The development of self-rated health during adolescence: an exploration of inter- and intra-cohort effects Can J Public Health 1999 90 90 94 10349213 10.1007/BF03404108 PMC6979667

[B41] Wade TJ Pevalin DJ Vingilis E Revisiting student self-rated physical health J Adolesc 2000 23 785 791 10.1006/jado.2000.0359 11161339

[B42] Vingilis ER Wade TJ Seeley JS Predictors of adolescent self-rated health: analysis of the National Population Health Survey Can J Public Health 2002 93 193 197 12050986 10.1007/BF03404999 PMC6979869

[B43] Vingilis ER Wade TJ Adlaf E What factors predict student self-rated physical health? J Adolesc 1998 21 83 97 10.1006/jado.1997.0131 9503077

[B44] Radloff LS The CES-D Scale: a self-report depression scale for research in the general population Appl psychol meas 1977 1 385 401 10.1177/014662167700100306

[B45] Zumbo BD Gadermann AM Zeisser C Ordinal versions of coefficients alpha and theta for Likert rating scales J Mod Appl Stat Methods 2007 6 21 29

[B46] Brener ND Kann L Kinchen SA Grunbaum JA Whalen L Eaton D Hawkins J Ross JG Methodology of the youth risk behavior surveillance system MMWR Recomm Rep 2004 53 1 13 15385915

[B47] Adlaf EM Bacglia-Boak A Beitchman JH Wolfe D The Mental Health and Well-Being of Ontario Students, 1991-2007: Detailed OSDUS Findings 2007 Toronto, ON: Centre for Addiction and Mental Health http://www.camh.net/Research/Areas_of_research/Population_Life_Course_Studies/OSDUS/OSDUHS2007_MentalHealth_Detailed_Final.pdf

[B48] Huebner ES Manual for the Multidimensional Students' Life Satisfaction Scale 2001 Columbia: University of South Carolina http://www.cas.sc.edu/psyc/pdfdocs/huebslssmanual.doc

[B49] Gilman R Huebner ES Laughlin JE A first study of the Multidimensional Students' Life Satisfaction Scale with adolescents Soc Indic Res 2000 52 135 160 10.1023/A:1007059227507

[B50] Cantril H Pattern of Human Concerns 1966 Piscataway, NJ: Rutgers University Press

[B51] Fayers P Machin D Quality of Life: The Assessment, Analysis and Interpretation of Patient-Reported Outcomes 2007 Chichester, West Sussex, England: John Wiley & Sons

[B52] Andrews FM Robinson JP Robinson JP, Shaver PR, Wrightsman LS, Andrews FM, Robinson JP Measures of subjective well-being Measures of Personality and Social Psychological Attitudes 1991 San Diego: Academic Press 61 110

[B53] Bowling A Measuring Health: A Review of Quality of Life Measurement Scales 2005 3 Maidenhead, England: Open University Press

[B54] Statistics Canada Ethnicity 2003 Ottawa, ON: Statistics Canada http://www.statcan.ca/english/concepts/definitions/ethnicity.htm

[B55] Schumacker RE Lomax RG A Beginner's Guide to Structural Equation Modeling 2004 2 Mahwah, NJ: Lawrence Erlbaum

[B56] Millsap RE Yun-Tein J Assessing factorial invariance in ordered-categorical measures Multivariate Behav Res 2004 39 479 10.1207/S15327906MBR3903_4

[B57] Jöreskog KG New developments in LISREL: analysis of ordinal variables using polychoric correlations and weighted least squares Qual Quant 1990 24 387 404 10.1007/BF00152012

[B58] Rigdon EE Ferguson CE Jr The performance of the polychoric correlation coefficient and selected fitting functions in confirmatory factor analysis with ordinal data J Mark Res 1991 28 491 497 10.2307/3172790

[B59] Muthén B Muthén L MPlus (version 5.2) 2008 Los Angeles, CA: Statmodel

[B60] Finney SJ DiStefano C Hancock GR, Mueller RO Non-normal and categorical data in structural equation modeling Structural Equation Modeling: A Second Course 2006 Greenwich, CT: Information Age Publishing 269 314

[B61] Beauducel A Herzberg PY On the performance of maximum likelihood versus means and variance adjusted weighted least squares estimation in CFA Struct equ modeling 2006 13 186 203 10.1207/s15328007sem1302_2

[B62] Yu CY Evaluating cutoff criteria of model fit indices for latent variable models with binary and continuous outcomes PhD thesis 2002 University of California, Department of Education

[B63] Hayduk LA Lisrel: Issues, Debates, and Stragies 1996 London: The Johns Hopkins University Press

[B64] McDonald RP Ho MHR Principles and practice in reporting structural equation analyses Psychol Methods 2002 7 64 82 10.1037/1082-989X.7.1.64 11928891

[B65] Thomas DR Hughes E Zumbo BD On variable importance in linear regression Soc Indic Res 1998 45 253 275 10.1023/A:1006954016433

[B66] Mackinnon DP Introduction to Statistical Mediation Analysis 2008 New York: Lawrence Erlbaum Associates

[B67] Mackinnon DP Dwyer JH Estimating mediated effects in prevention studies Eval Rev 1993 17 144 158 10.1177/0193841X9301700202

[B68] Rubin DB Multiple Imputation for Nonresponse in Surveys 1987 New York: Wiley

[B69] SAS Institute Statistical Analysis Software (version 9.2) 2005 Cary, NC: Author

[B70] Allison PD Missing Data 2002 Thousand Oaks, CA: Sage

[B71] Enders CK Hancock GR, Mueller RO Analyzing structural equation models with missing data Structural Equation Modeling: A Second Course 2006 Greenwich: Information Age Publishing 313 342

[B72] Gilman R Validation of the Multidimensional Students' Life Satisfaction Scale with adolescents PhD thesis 1999 University of South Carolina, Department of Psychology

[B73] Huebner ES Gilman R An introduction to the Multidimensional Students' Life Satisfaction Scale Soc Indic Res 2002 60 115 122 10.1023/A:1021252812882

[B74] Huebner ES Laughlin JE Ash C Gilman R Further validation of the Multidimensional Students' Life Satisfaction Scale J Psychoeduc Assess 1998 16 118 134 10.1177/073428299801600202

